# PD02 Budget Impact Analysis: A Challenge To Incorporating Medications For Ultrarare Diseases In The Brazilian Healthcare System

**DOI:** 10.1017/S0266462324002721

**Published:** 2025-01-07

**Authors:** Marisa Santos, Daniel da Silva Pereira Curado, Juliana Machado-Rugolo, Denis Satoshi Komoda, Mônica Aparecida de Paula De Sordi, Alan Francisco Fonseca, Juliana Tereza Coneglian de Almeida, Silvana Molina Lima, Silke Anna Theresa Weber, Jean-Eric Tarride, Lehana Thabane, Marilia Mastrocolla de Almeida Cardoso, Luis Gustavo Modelli de Andrade

## Abstract

**Introduction:**

The decision-making process for incorporating technologies for ultrarare diseases (URD) has been a challenge for health technology assessment agencies worldwide. These challenges have been presented in debates about the budget impact of incorporating technologies for URD. This is an important issue because there are other dimensions of the economic and social impact of URD that require consideration.

**Methods:**

Data were extracted from National Committee for Health Technology Incorporation (CONITEC) reports (2012 to 2022) on technologies for the treatment of URD in Brazil. Diseases were classified using an epidemiological criterion or Orphanet consultation (prevalence ≤1 per 50,000 inhabitants). Variables included eligible patient count, population estimation method, incremental impact values for one and five years, and diffusion rate in the first and fifth year. Univariate logistic regression was used to adjust the relationship between the budget impact analysis and the final recommendation, considering factors associated with incorporation in univariate regression and p-values less than 0.10 in a multivariate regression.

**Results:**

Among 53 reports, 48 percent exclusively employed the epidemiological approach for incremental impact assessment population estimation, rising to 69.5 percent when combined with measured demand. Population data were nearly evenly sourced from national and international platforms, with the UK, the USA, and multicenter studies being the most cited internationally. Notable differences were found between favorable and unfavorable CONITEC recommendations, with lower values being associated with incorporation. Market share diffusion rates favored the option of 100 percent diffusion in both the first year and the cumulative five years. The analysis highlighted the influence of demand characteristics and technology type on the budget impact value over one and five years.

**Conclusions:**

The study found that budget impact data significantly influenced the final recommendation for technology incorporation, indicating a criterion favoring technologies with a lower budget impact. However, requester characteristics and technology type also played a role in the decision-making process, suggesting that additional factors influence recommendations.

